# Music and trust formation: a scoping review of neurobiological convergence and research priorities

**DOI:** 10.3389/fpsyg.2026.1731470

**Published:** 2026-05-08

**Authors:** Cairang Guanque, Shilei Huang, Xiyuan Wang, Xue Lei, Keyu Zhai, Chengyang Han, Frank Krueger

**Affiliations:** 1 Jinghengyi School of Education, Hangzhou Normal University, Hangzhou, China; 2 Institute of Psychological and Brain Sciences, Liaoning Normal University, Dalian, China; 3 Key Laboratory of Brain and Cognitive Neuroscience, Dalian, China; 4 School of Management, Zhejiang University of Finance and Economics, Hangzhou, China; 5 School of Graduate Studies, Lingnan University, Hong Kong, Hong Kong SAR, China; 6 Zhejiang Philosophy and Social Science Laboratory for Research in Early Development and Childcare, Hangzhou Normal University, Hangzhou, China; 7 School of Systems Biology, George Mason University, Fairfax, VA, United States

**Keywords:** music psychology, preparatory optimization, scoping review, social neuroscience, trust formation

## Abstract

**Introduction:**

Trust is a cornerstone of human cooperation and social cohesion, while music is a universal phenomenon that promotes prosocial behavior and social bonding. Both music and trust processes engage overlapping neurobiological systems, yet their potential relationship remains largely unexplored.

**Methods:**

We conducted a scoping review to systematically map existing research on music’s influence on trust formation, identify conceptual and methodological gaps, and characterize the current evidence base. The analysis included 15 empirical studies across interpersonal, clinical, robotic, and institutional trust contexts. We further performed a hypothesis-generating convergence analysis of neurochemical, neuroimaging, and electrophysiological findings from the broader music and trust literatures.

**Results:**

The review revealed sparse but suggestive evidence that musical interventions may influence trust-related outcomes. Convergence analysis identified shared mechanisms in dopaminergic reward pathways, the salience and default-mode networks, and EEG markers of salience and affect. Importantly, these patterns are interpreted as theoretical and exploratory, rather than direct evidence linking music and trust.

**Discussion:**

Based on these findings, we propose the Preparatory Optimization Hypothesis, which posits that music may not directly induce trust, but instead primes affective, cognitive, and social systems to support its formation. This work extends music psychology beyond emotion and prosociality to include foundational social-cognitive processes, offering a testable framework for future interdisciplinary research at the interface of music and trust.

## 1 Introduction

This introduction establishes the foundational concepts necessary for understanding music-trust relationships. We first define trust and its neurobiological foundations, examine music’s prosocial effects and neural mechanisms, and conclude by presenting the rationale for this scoping review and our theoretical framework development approach.

### 1.1 Trust: definition, measurement, and neurobiological foundations

Trust is the cornerstone of human civilization, enabling cooperation, social cohesion, and economic prosperity across diverse cultural contexts (Knack and Keefer, 1997). It is defined as an individual’s readiness to accept vulnerability to another party, driven by expectations that this party will act in ways that benefit the trustor, even when the trustor cannot monitor or control the other’s actions (Mayer et al., 1995). This capacity represents a fundamental evolutionary adaptation that enabled early humans to form cooperative alliances, share resources during scarcity, and coordinate group activities essential for survival (Cosmides and Tooby, 2005). Trust manifests across multiple domains, including human-human trust between individuals (McAllister, 1995; Birkhäuer et al., 2017), human-system/organization trust in institutions (Rothstein and Teorell, 2008), human-AI/robot trust in autonomy/automation (Hancock et al., 2011), and human-self-trust in one’s abilities (Stankov and Lee, 2008).

Trust can be measured through multiple validated approaches, which are critical for evaluating interventions. Self-report questionnaires assess general trust propensity using established scales such as the General Trust Scale (Yamagishi and Yamagishi, 1994), the Trust in Close Relationships scale (Rempel et al., 1985), and the Interpersonal Trust Scale (Rotter, 1967), while behavioral economic exchange games directly reveal trust behaviors through decision-making (Camerer, 2009). Specifically, the two-person interpersonal trust (investment) game measures willingness to be vulnerable through financial risk-taking (Berg et al., 1995). Cooperation-based economic game paradigms such as the Prisoner’s Dilemma (Flood and Dresher, 1950) and the Public Goods Game (Isaac et al., 1994) assess trust through cooperative decisions and collective resource contributions. Neuroscience methods can capture neurobiological markers of trust-related motivational, emotional, and cognitive components through hormonal and neurotransmitter measures (Kosfeld et al., 2005), functional magnetic resonance imaging (fMRI) (Krueger et al., 2007), and electroencephalography (EEG) (Wang et al., 2017), including event-related potentials (ERPs) (Tortosa et al., 2013).

The neuroscience of trust involves complex interactions across multiple neurobiological systems (Krueger, 2021). Neurochemically, trust formation has been associated, for example, with oxytocin-related processes involved in social behavior and cooperation (Kosfeld et al., 2005), dopamine for reward reinforcement (Delgado et al., 2005), and testosterone modulation for competitive evaluation during social interactions (Boksem et al., 2013). Neural circuits engage four large-scale networks: salience network (SAN, associated with betrayal aversion), reward network (RWN, involved in reward anticipation), default-mode network (DMN, involved in social cognition), and central-executive network (CEN, involved in strategic planning) (Krueger and Meyer-Lindenberg, 2019). Electrophysiologically, trust processing reveals frontal alpha asymmetry EEG patterns that reflect approach-avoidance behavioral tendencies during social decision-making (Tortosa et al., 2013), while ERP provides insights into trust-related decision-making through outcome evaluation signals such as the P3 component during trust games (Boudreau et al., 2009). While trust represents a fundamental psychological process for human cooperation (Tomasello, 2009), other uniquely human phenomena, such as music, may contribute to social bonding and interpersonal connection.

### 1.2 Music: evolution, prosocial effects, and neural mechanisms

Music represents one of humanity’s most universal phenomena across all societies throughout history (Savage et al., 2015). Archaeological evidence of ancient instruments dating back tens of thousands of years (Conard et al., 2009) indicates the long-standing presence of music in human societies and has been interpreted as consistent with the possibility that music may have contributed to social cohesion (Dunbar, 2012), with music-making activities such as singing, drumming, and dancing being deeply embedded in cultural rituals that require social coordination (McNeill, 1995). This ubiquity suggests that music evolved as a fundamental mechanism for creating and strengthening social bonds through interpersonal synchrony (Hove and Risen, 2009), shared emotional experiences (Juslin and Västfjäll, 2008), and group coordination (Cross, 2001), which are essential for early human survival and cooperation (Dunbar, 2012).

Research suggests that musical engagement promotes prosocial behaviors, including cooperation and helping behaviors, through collective participation (Kirschner and Tomasello, 2010). Synchronous musical activities—such as singing, dancing, or playing together—particularly strengthen social bonding and willingness to cooperate (Wiltermuth and Heath, 2009), with effects that extend beyond the musical context into general interpersonal trust and collaboration (Launay et al., 2016). Contemporary music therapy research documents applications for enhancing social skills across diverse populations, from improving social communication in children with autism (Geretsegger et al., 2014) to reducing isolation in elderly populations (Särkämö et al., 2014), demonstrating music’s broad capacity for fostering social connection (Wheeler, 2015).

The neuroscience of music reveals extensive engagement of brain systems underlying social bonding and emotional regulation (Koelsch, 2014). Neurochemically, music activates reward systems through the release of dopamine (Salimpoor et al., 2011), reduces stress by regulating cortisol levels (Chanda and Levitin, 2013), and promotes social bonding through the release of oxytocin during group musical activities (Keeler et al., 2015). Neural circuits involved in music processing overlap significantly with core domain-general large-scale networks, including RWN for pleasure and motivation (Zatorre, 2015), DMN for emotional integration (Wilkins et al., 2014), and SAN for attention and emotional significance (Koelsch, 2014). Electrophysiologically, music influences brain oscillations associated with emotional regulation, particularly alpha band asymmetries linked to approach behaviors (Arjmand et al., 2017), while ERP reveals neural responses to musical structure and emotional processing during aesthetic experiences (Koelsch, 2014).

Given music’s power to evoke emotions, synchronize behavior, and promote social relationships, these observations raise a compelling question: can music specifically enhance trust between individuals, and what neural mechanisms underlie this potential effect?

### 1.3 Scoping review rationale and theoretical framework development

While research has extensively investigated the effects of music on emotions (Juslin and Västfjäll, 2008) and social bonding (Dunbar, 2012), the specific relationship between music and trust repre-sents an emerging field requiring systematic mapping in social neuroscience. The current literature lacks a comprehensive understanding of the scope, nature, and methodological approaches used to investigate music-trust relationships, representing an unmapped research landscape given the importance of both phenomena for human cooperation and social functioning (Nowak, 2006). This scoping review addresses this knowledge gap by mapping the nascent research landscape of music-trust relationships and identifying priorities for systematic investigation (Peters et al., 2020). Our approach recognizes that this field requires foundational mapping before establishing definitive mechanisms (Levac et al., 2010). Throughout the manuscript, relationships between variables are described cautiously, reflecting the predominantly correlational and heterogeneous nature of the available evidence.

Our theoretical contribution centers on the *Preparatory Optimization Hypothesis*, a novel framework proposing that music may influence trust indirectly by creating conditions that support subsequent trust formation, rather than directly inducing trust mechanisms. This hypothesis emerges from a hypothesis-generating convergence analysis of shared neurobiological systems underlying both processes (Kober et al., 2008), examined through the neuropsychoeconomic T-R-U-S-T framework (conceptualizing trust as a dynamic interplay between Treachery, Reward, Uncertainty, Strategy, and Trustworthiness) (Krueger and Meyer-Lindenberg, 2019). Given the limited direct evidence linking music and trust, we adopt a convergence approach that systematically examines how both processes have been shown to engage overlapping neurobiological systems to identify potential mechanisms and generate testable predictions (Poldrack, 2006). Importantly, this convergence analysis is theoretical and exploratory and does not constitute direct empirical evidence linking music and trust. This methodology is appropriate for emerging fields where theoretical development must precede extensive empirical validation. By identifying preliminary evidence patterns, highlighting potential shared neural mechanisms, and proposing testable theoretical frameworks, this review provides foundational knowledge for systematic research programs that can address current gaps through targeted experimental validation, cross-cultural investigation, and mechanistic studies (Henrich et al., 2010).

## 2 Mapping the research landscape

This section presents our systematic scoping review methodology and findings. We first describe our approach for identifying and evaluating relevant studies, then analyze the research activity and conceptual patterns across different trust relationship types, and conclude by identifying critical research gaps and methodological development needs.

### 2.1 Scoping review methodology

This scoping review followed the methodological framework developed by Arksey and O'Malley (2005), which provides a systematic approach for mapping research activity in emerging fields, identifying key concepts and research gaps, and determining priorities for future investigation (Levac et al., 2010). The scoping review methodology is particularly appropriate when the research base is heterogeneous and the primary aim is to map the breadth of evidence rather than assess quality or synthesize findings (Daudt et al., 2013). This followed the PRISMA extension for Scoping Reviews (PRISMA-ScR) guidelines (Tricco et al., 2018) with a predefined protocol, including data sources, search strategy, inclusion criteria, and screening methods (Peters et al., 2020).

#### 2.1.1 Data sources and search strategy

Literature searches were conducted across six databases: PubMed, Web of Science, Scopus, PsycINFO, IEEE Xplore, and Google Scholar. Searches covered publications from 1990 to May 15, 2025, limited to English-language studies. A Boolean search strategy combined three key term categories: (1) music terms: “music,” “musical,” “song,” “melody,” “therapy,” “synchronization,” “emotional music”; (2) trust terms: “trust,” “trustworthiness,” “social bonding,” “cooperation,” “trust game,” “investment game,” “prisoner’s dilemma,” “public goods game”; and (3) neuro-biological terms: “neurobiology,” “neurochemical,” “hormones,” “neurotransmitters,” “fMRI,” “functional near-infrared spectroscopy (fNIRS),” “positron emission tomography (PET),” “EEG,” and “ERP.” These three term categories were combined using Boolean operators and adapted as needed for each database to account for differences in indexing and search functionality. Google Scholar searches were limited to the first 200 results, based on relevance ranking and consistent with common practice due to decreasing precision beyond this range. Reference lists of included studies underwent manual screening. Prior to screening, systematic deduplication was performed using Zotero software (Corporation for Digital Scholarship, 2025). Although this restriction may have excluded some relevant studies, Google Scholar was used as a supplementary source alongside multiple databases to enhance the overall thoroughness of the search.

#### 2.1.2 Inclusion and exclusion criteria

Inclusion criteria encompassed empirical studies (including experimental, observational, and qualitative designs), systematic reviews, and meta-analyses published in peer-reviewed English-language journals. Studies must explicitly investigate music’s influence on trust using validated measures. Eligible populations included both healthy and clinical samples across diverse contexts (e.g., interpersonal, clinical, human–robot, and organizational settings). The review focused on studies involving direct musical interventions or exposure to musical stimuli; purely correlational studies without a music manipulation were not included. Music definition included melodic, harmonic, or song-based content, including singing, instrumental music, or musical prosody. Studies examining synchrony without an explicit musical component were excluded, as the present review focuses on music as the primary variable of interest rather than its underlying components in isolation. Trust outcomes encompassed interpersonal trust, human-AI/robot trust, institutional trust, therapeutic trust, and self-trust. Measurement approaches required validated paradigms, including economic trust games, interpersonal trust scales, behavioral assessments, or physiological indicators, as well as self-report measures of perceived trustworthiness, which were included as related but conceptually distinct constructs. Studies with and without explicit comparator conditions were included to reflect the heterogeneity of methodological approaches in this emerging field. These inclusion criteria are summarized using a PICOS framework (Table 1) (Tricco et al., 2018).

**Table 1:** PICOS framework for study inclusion criteria.

Component	Description
Population (P)	Human participants across diverse populations, including healthy adults and clinical samples, as well as interaction contexts such as interpersonal, clinical, human–robot, and organizational settings.
Intervention/exposure (I)	Musical stimuli or music-based activities, including active forms (e.g., singing, music-making, music therapy) and passive forms (e.g., listening, background music, musical prosody).
Comparator (C)	Studies with and without explicit comparator conditions were included. Where present, comparators included non-musical conditions (e.g., silence, speech, non-musical tasks) or alternative musical conditions.
Outcome (O)	Measures related to trust, including behavioral measures (e.g., trust games), self-report trust scales, and perceived trustworthiness as a related but conceptually distinct construct.
Study design (S)	Empirical studies including experimental, observational, and qualitative designs, as well as systematic reviews and meta-analyses published in peer-reviewed journals.

Exclusion criteria eliminated non-empirical papers, grey literature, studies examining music and trust as separate variables without investigating their relationship, studies investigating only isolated rhythmic elements, research examining general prosocial behavior without specific trust measures, and studies without clearly defined trust outcome measures.

#### 2.1.3 Study selection process

**Figure 1:**
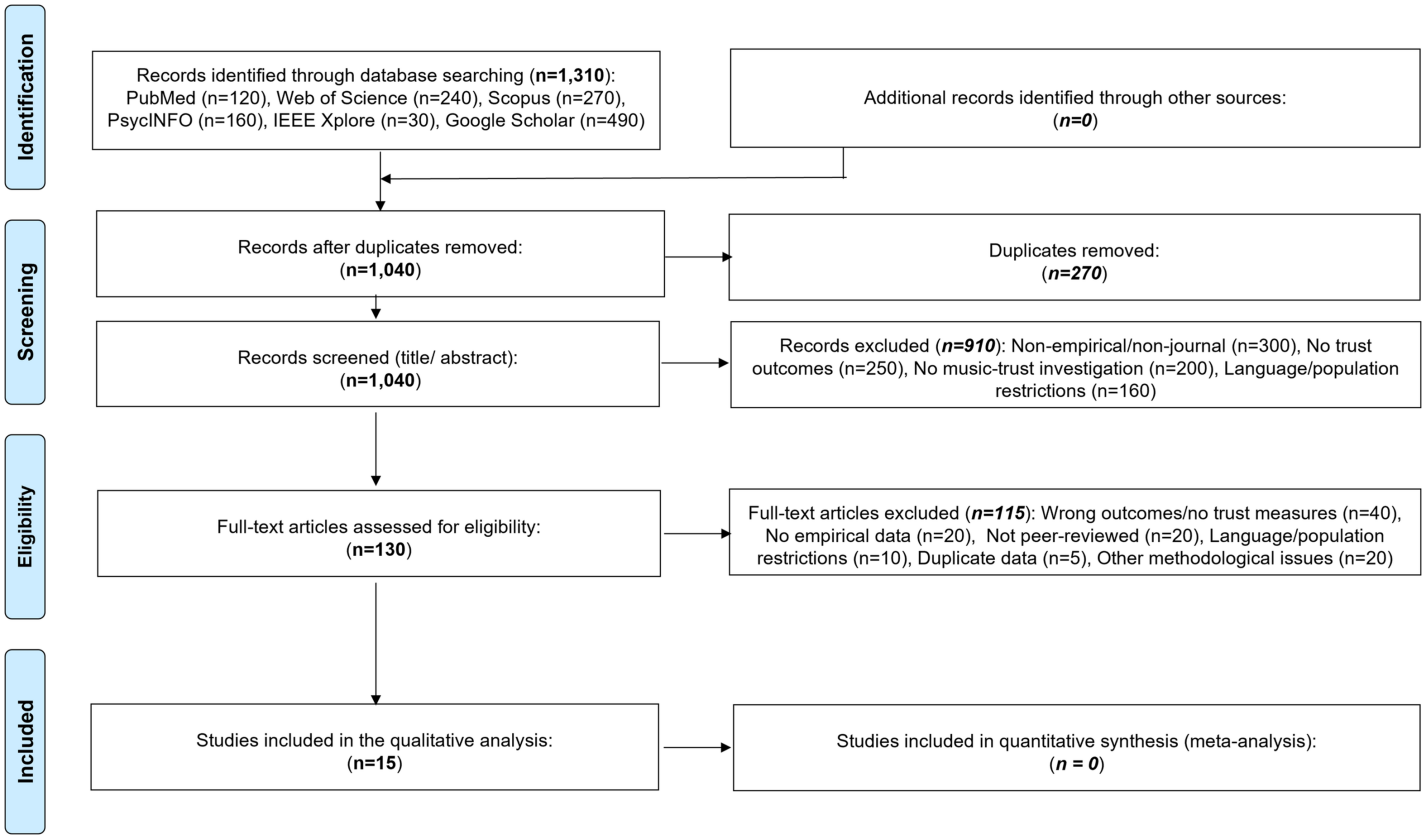
PRISMA-ScR flow diagram showing source selection process for scoping review of music’s influence on trust (15 sources identified from 1,040 records).

Following systematic database searches and removal of duplicates, 1,040 unique records underwent a two-stage screening process. Screening was conducted by two reviewers with experience in systematic and scoping review methodology. Initial screening involved independent title and abstract review, followed by full-text assessment against inclusion and exclusion criteria. Discrepancies were resolved through discussion and consensus, with involvement of a senior author when necessary. This process resulted in 15 studies meeting all criteria for qualitative synthesis (Figure 1). Given the exploratory scope of this scoping review, formal inter-rater reliability statistics (e.g., Cohen’s kappa) were not calculated, consistent with methodological guidance emphasizing comprehensive coverage over agreement metrics in emerging research fields (Levac et al., 2010; Tricco et al., 2018).

#### 2.1.4 Data extraction and quality assessment

A standardized data extraction form collected study characteristics (authors, year, design, sample size, demographics), musical intervention details (type, genre, delivery method, duration), trust variables (target, measurement approach, instruments), and outcomes (primary and secondary findings, overall effects). Due to substantial heterogeneity in study designs, interventions, measures, and populations, a meta-analysis was not feasible. Narrative synthesis analyzed findings by trust type and methodology. Most evidence reflects correlational rather than causal relationships due to limited experimental manipulation of proposed mechanisms.

### 2.2 Research activity and conceptual mapping

#### 2.2.1 Research activity mapping

Research activity examining music-trust relationships spans from 1988 to 2023, encompassing 15 empirical studies with approximately 1,626 + participants across diverse populations. Studies are distributed across multiple geographic regions, including the United States, Austria, Turkey, China, and Israel. The included studies were predominantly conducted in Western contexts, particularly in the United States, with more limited representation from non-Western regions such as China, Turkey, and Israel, highlighting an imbalance in the current evidence base. Sample sizes ranged from 24 to over 460 participants, with most studies utilizing between-subjects experimental designs, while others employed within-subjects, mixed designs, or observational approaches (Table 2).

**Table 2:** Scoping review of music-trust studies: research characteristics, interventions, and patterns across trust relationship types (*N* = 15 studies, 1990–2025).

Authors (Year)	Study design	N	Population	Musical engagement	Trust Target	Trust measurement (dependent variable)	Music type genre	Main finding	Effect
Human-human trust									
Anshel and Kipper (1988)	EXP | B-S: 2 music x 2 activity, 4 groups	96(24 per group)	Israeli adults; age: 32.0 ± 4.2; 100% male	B-F: (1) group singing (music + activity), (2) listening to music (music + no activity), (3) poetry reading (no music + activity), and (4) film viewing (no music + no activity)	Human-Stranger (person sitting to right)	Direct-Self-Report (18 items, only Character and Dynamism subscales, excluding expertness) + Direct-Behavior (Prisoner’s Dilemma Game, 5 rounds)	Popular Israeli songs (piano/trumpet accompaniment)	Music (singing or listening) increased self-reported trust but not behavioral trust; active participation (singing or poetry reading) increased behavioral trust but not self-reported trust; no interaction effects	+
Wiltermuth and Heath (2009) (Study 3 Only)	EXP | B-S: 4 groups (cluster randomization by groups of 3)	105 (3 per group, 35 groups)	U.S. adults; age: 21.0 ± 2.0; 34% male	B-F: (1) synchronous singing and moving, (2) synchronous singing (no moving), (3) asynchronous singing and moving, and (4) control (no singing, no moving)	Human-Stranger (other group members)	Direct-Self-Report (“How much did you trust the other participants going into the exercise?,” 7-point Likert scale) + Direct-Behavior (Public Goods Game)	“O Canada” (anthem)	Synchrony increased behavioral trust more than self-reported trust (marginally significant); behavioral trust persisted over rounds in synchronous conditions but declined in asynchronous conditions, mediated by team feelings	±
Riedl et al. (2017)	EXP | B-S:2 groups	30 (15 per group)	Austrian graduate and doctoral students; age: 26.6 ± 57; 100% male	B-F: (1) background music listening, and (2) no music during trust game	Human-Virtual (faces and nicknames)	Direct-Self-Report (trustworthiness rating as precursor to trust, 7-point Likert Scale) + Direct-Behavior (Iterated Trust Game, 40 rounds) + Physiological (Blood Oxytocin)	Schubert’s “Marche Militaire” D major (classical martial)	No effect of music on oxytocin, trust behavior, or trustworthiness; oxytocin-trustworthiness correlation only in no-music condition	0
Rubin et al. (2001)	EXP | B-S:6 groups	243 (group sizes ranged from 16 to 70 based on self-reported music preferences)	U.S. college students; age: 21.6 ± 4.7; 47.3% male	B-F: Listening to six preferred popular music genres: (1) alternative/alternative pop, (2) classic rock/adult contemporary, (3) dance/rhythm and blues/soul, (4) country western, (5) hard rock/heavy metal, and (6) rap	Human-others (general interpersonal distrust)	Direct-Self-Report (Rosenberg’s Faith in People Scale, 5-items, measuring generalized trust/distrust in others)	preferred popular music genres (alternative/alternative pop, classic rock/adult contemporary, dance/rhythm and blues/soul, country western, hard rock/heavy metal, rap)	Rap listeners showed significantly higher distrust compared to alternative, classic rock/adult contemporary, and country listeners, but not compared to dance/soul or heavy metal listeners	±
Human-robot trust									
Savery et al. (2019)	EXP | B-S:2 groups	24 (12 per group)	U. S. undergraduate students; age & gender not reported	B-F: (1) Music-driven prosody (heard Shimi’s non-linguistic, music-driven emotional vocalizations), and (2) text-to-speech group (heard traditional text-to-speech audio using CereProc’s Meghan voice)	Human–Robot (user–robot)	Direct-Self-Report (Trust Perception Scale–HRI, 40-items, 0–100%)	MIDI-controlled non-linguistic vocalizations (emotional musical phrases driving synthesized phonemes and electronic sounds)	Music-driven emotional prosody significantly increased trust in the robot compared to traditional text-to-speech audio	+
Savery et al. (2021b)	EXP | B-S:3 groups	46 (approx. 15 per group)	Adults recruited online via Prolific; age: 25.0 ± 7.0; gender not reported	B-F: Participants interacted with virtual robotic arm during pattern recognition task; arm provided feedback via (1) Emotional Musical Prosody (EMP) audio + gestures, (2) single-pitch audio + gestures, and (3) estures-only (no audio)	Human–Robot (user–robot)	Direct-Self-Report (Schaefer Trust Perception Scale-HRI, 14 items, 0–100%) + Direct-Behavior Direct-Behavioral (% agreement with robot’s suggestions during pattern task)	Singer-improvised non-linguistic vocalizations (1–15 s phrases) emotion-tagged to Geneva Emotion Wheel; 4 emotions used: joy, shame, sadness, anger	EMP increased self-reported trust vs. single-pitch and no-audio; no effect on behavioral trust	±
Savery et al. (2021a)	EXP | B-S:3 groups	153 (approx. 51 per group)	Adults recruited via MTurk and Prolific; age: 37 ± 12; 59% male	B-F: Interaction with three virtual robotic arms performing collaborative tasks with (1) gesture only (no audio), (2) single emotional musical prosody voice, and (3) multiple emotional musical prosody voices	Human-Robot (user-robot group interaction)	Direct-Self-Report (Schaefer Trust Perception Scale-HRI, 14 items, 0–100%)	Emotional Musical Prosody (music-derived prosody phrases expressing positive emotions: admiration, contentment, compassion)	Both single and multiple emotional musical prosody voice increased trust ratings compared to the no audio condition, with no significant difference between the two prosody conditions	+
Liu et al. (2023)	EXP | W-S:4 conditions	40	U.S. university students; age: 19–27, M ± SD not reported; 52% male	W-F: (1) natural voice human (non-lexical vocalizations [e.g., “Woohoo” for happiness, “Argh” for anger, crying for sadness, screams for fear, gasps for surprise]), (2) synthesized voice same vocalizations but processed with robotic/metallic effects [using Flanger and Flangus effects to make them sound more traditionally “robotic”], (3) musical sound (non-linguistic musical motifs/earcons [e.g., piano minor chords for sadness, tremolo strings for fear, upbeat sounds for happiness, distorted guitar for anger]), and (4) no sound (gestures only, robot gestures without any auditory component [baseline condition])	Human–Robot (user–robot)	Indirect-Self-Report (trustworthiness as precursor to trust: “How trustworthy is the voice/gesture?”; 7-Point Likert scale)	abstract musical sound effects specifically designed for emotional expression in human-computer interaction contexts	Natural voice non-speech sounds (e.g., ‘woohoo’, ‘argh’) significantly enhanced robot trustworthiness (precursor to trust) compared to musical sounds and marginally compared to synthesized voice; musical sounds showed the lowest trustworthiness ratings among all conditions including gesture-only baseline	−
Human-professional trust									
Silverman (2014)	EXP | B-S:4 groups (cluster-randomized)	96 (about 24 per group)	U.S. acute psychiatric inpatients (mixed diagnoses: bipolar disorder, major depressive disorder, schizoaffective disorder, schizophrenia); age: 35.3 ± 14.3; 49% male	B-F: (1) live educational music therapy (MT) (“Runaway Train” by Soul Asylum - performed live on acoustic guitar), (2) recorded educational music therapy (“Runaway Train” by Soul Asylum - recorded version played), (3) Education without music (no music, discussion only), and (4) recreational MT (Rock and roll bingo - various rock songs played from portable stereo)	Human-Professional (music therapist)	Direct-Self-Report (Wake Forest Physician Trust Scale, 10 items, 4 subscales: fidelity, competence, honesty, global)	“Runaway Train” by Soul Asylum (alternative rock); various rock songs for bingo	No differences in total trust scores; live educational MT produced significantly higher therapist competence ratings compared to recorded educational MT (competence is one of four trust subscales)	±
Silverman (2015)	EXP | B-S:4 groups (cluster-randomized)	130 (approx. 32 per group)	U.S. detoxification inpatients; age: 39.4 ± 14.5; 50% male	B-F: (1) live educational music therapy (MT) (“Runaway Train” by Soul Asylum - performed live by therapist on acoustic guitar), (2) recorded educational MT (“Runaway Train” by Soul Asylum—recorded version played), (3) education without music (no music, discussion only), and (4) recreational MT (Rock and roll bingo—various rock songs played from portable stereo)	Human-Professional (music therapist)	Direct-Self-Report (Wake Forest Physician Trust Scale, Likert scale)	“Runaway Train” by Soul Asylum (alternative rock)	No differences between groups on trust	0
Rodgers-Melnick et al. (2019)	EXP | W-S:1 condition (5 levels)	28 (only 24 completed baseline measures for trust)	U.S. adolescents/young adults with sickle cell disease; age: 21.0 ± 1.4; 50% male	W-F: BEATS (Build, Educate, Advance, Transition, in Sickle cell disease) music therapy program (fill-in-the-blank songwriting, song stories, instrumental improvisation, group drumming) delivered at 3, 6, 9, and 12 months; measurements at baseline, 3, 6, 9, and 12 months	Human-Professional (healthcare provider)	Direct-Self-Report (Wake Forest Trust in the Medical Profession Scale, 5 items)	Therapeutic drumming, original songwriting (genre not specified), instrumental improvisation	No changes in trust toward healthcare providers over 12 months	0
Human-Self Trust									
Zhou et al. (2023)	EXP | W-S:1 condition (8 levels)	32 (from DEAP dataset) + unspecified number from DREAMER dataset + (validation experiment)	No demographic information (age, gender, or country) provided	W-F: eight emotional music conditions presented as audiovisual stimuli (music synchronized with video clips = 40) representing (1) fear, (2) confidence, (3) joy, (4) anger, (5) disgust, (6) passion, (7) relaxation, and (8) sadness	Human-self (participant’s emotional state)	Direct-Physiological EEG-based Emotion Recognition (EER) model utilizing Power Spectral Density analysis to identify 20 distinct emotional states (including trust) based on a previously validated semantic emotion framework.	Emotion-based music content (not genre-specific)	EER model achieved 96.47% accuracy in identifying 20 emotional states including trust; joy-type music transformed trust emotions into surprise emotions (44.44% probability), suggesting music induces emotional state transitions rather than simple enhancement	+
Yüksel et al. (2023)	EXP | B-S: 2 groups	100 (50 per group)	Turkish adults; age: 26.0 ± 11.2; 22% male	B-F: Weekly listening: (1) pre/post-lingually deafened cochlear implant (CI) recipients (CI: 5.9 ± 1.1 h), and (2) normal hearing [NH] controls (NH: 13.6 ± 1.5 h)	Human-self (participant’s emotional state)	Direct-Self-Report Trust as 1 of 28 emotions; 5-point Likert scale (frequency of experiencing trust in reaction to music)	Personally meaningful music (varied genres)	CI group reported high trust levels, significantly more than NH group	+
Human system/organization trust									
Schmidmaier et al. (2020)	EXP | W-S:1 condition (19 levels)	39	Adults recurited online; age: 18–64, M ± SD not reported; 56% male	W-F: 19 audio patterns (16 main experimental patterns varying in: 4 melody shapes [ascending, valley-shaped, arch-shaped, descending], 2 pitch levels [high/low], 2 modes [major/minor], 2 tempos [fast/slow]; and 3 additional patterns: Android melody simulation, iOS message sound simulation, test pattern for sound style preference)	Human–System (technology interaction)	Indirect-Self-Report (Perceived trustworthiness of audio patterns [precursor to trust]; “I found this audio pattern trustworthy”; 4-point Likert scale)	Synthesized and piano short melodies	Ascending melodies rated more trustworthy (precursor of trust) than descending; major mode more trustworthy than minor	+
Meng et al. (2023)	OBS | WS: cross-sectional, survey-based	464	Chinese (Shanghai) supermarket customers; age: 34.8 ± 9.5; 40% male	W-F: Pleasantness of store music	Human–System (customer-store)	Indirect-Self-Report (perceived store trust)	Ambient background store music	Pleasantness of store music enhanced customer engagement, indirectly increasing customer trust in the store via mediation	+

EXP, experimental; OBS, observational; B-S, between-subjects; WS, within-subjects; age: M ± SD; B-F, between-factor; W-F, within-factor; +, positive effect; ±, mixed effect; 0, null effect; −, negative effect.

The research landscape reveals diverse findings across contexts and methodologies. Studies reported variable outcomes, including positive effects (7 studies), mixed or partial effects (4 studies), null effects (3 studies), and negative effects (1 study), indicating that music-trust relationships are highly context-dependent and methodologically sensitive. Studies employed diverse trust measurement approaches: self-report measures (14 studies), behavioral measures through economic games (4 studies), and physiological measures including oxytocin levels and EEG-based emotion recognition (2 studies).

Musical interventions varied significantly: active participation (3 studies), passive listening (12 studies), music therapy interventions (3 studies), and background/ambient music (2 studies). Musical genres included popular music (4 studies), computer-generated compositions (5 studies), emotion-based selections (7 studies), and classical, culturally specific, and therapeutic music.

#### 2.2.2 Conceptual categories and research patterns

The studies were organized into five categories based on trust relationship type (Table 2), revealing distinct patterns across contexts where trust mechanisms vary fundamentally by target—*interpersonal trust* relies on social cues, *human-robot trust* involves attributions of intelligence, *professional trust* centers on competence, *self-trust* relates to emotional regulation, and *system/organization trust* depends on institutional integrity and performance. Importantly, the overall pattern of findings is mixed, with studies reporting positive, mixed, null, and negative effects, highlighting the context-dependent nature of music–trust relationships.

To improve conceptual clarity, musical interventions across studies can be categorized along several dimensions. First, music engagement may be active (e.g., singing, joint music-making, music therapy participation) or passive (e.g., listening to recorded or background music). Second, music may function as a focal component of the interaction (e.g., as a primary communicative signal) or as a contextual/background element. Third, the relationship between music and the trust context varies, with music being experienced by the trustor, the trustee, or both parties simultaneously. Finally, some studies assess responses to musical or auditory stimuli (e.g., perceived trustworthiness of sounds), which are conceptually distinct from behavioral trust, defined as observable trust-related decisions involving interpersonal vulnerability (e.g., decisions in trust games), a distinction that is further examined in Section 2.2.3.

Human-Human Trust (*n* = 4) revealed variable findings with important boundary conditions. Anshel and Kipper (1988) found that music increased self-reported trust but not behavioral trust, while active participation increased behavioral trust but not self-reported trust, indicating music influences different trust dimensions through distinct mechanisms. Wiltermuth and Heath (2009) demonstrated that synchrony was more critical than music, with synchronous singing enhancing behavioral trust through increased “team feelings.” Riedl et al. (2017) found no effects of music on trust behavior, perceived trustworthiness, or oxytocin levels, with correlations between oxytocin and trustworthiness observed only in the no-music condition. Rubin et al. (2001) found that musical preferences influence baseline trust levels, with rap listeners showing significantly higher distrust than listeners of other genres.

Human-Robot Trust (*n* = 4) revealed variable effectiveness depending on the implementation approach. Research in this category indicates two studies found positive effects: Savery et al. (2019) showed that music-driven emotional prosody significantly increased trust compared to traditional text-to-speech audio, while Savery et al. (2021a) found that both single and multiple emotional musical prosody voices increased trust ratings compared to gesture-only conditions. However, one study showed mixed results (Savery et al., 2021b), where emotional musical prosody increased self-reported trust but did not affect behavioral trust. Additionally, Liu et al. (2023) found musical sounds decreased robot trustworthiness compared to natural voice non-speech sounds, indicating that music may be less effective—or even detrimental—when it conflicts with expectations of naturalistic social communication. Research patterns suggest variability based on using music as a medium for emotional communication rather than mere background accompaniment, highlighting that both positive and negative effects depend on how music is integrated into the interaction context.

Human-Professional Trust (*n* = 3) indicated limited effectiveness across clinical settings. Silverman (2014) found no overall trust differences among acute psychiatric inpatients but showed live educational music therapy increased therapist competence (i.e., trust subcomponent) ratings compared to recorded delivery. Silverman (2015) found no differences between music therapy conditions on trust among detoxification inpatients. Rodgers-Melnick et al. (2019) found no significant changes in trust toward healthcare providers over 12 months of music therapy intervention in adolescents with sickle cell disease.

Human-Self Trust (*n* = 2) indicated potential positive patterns through emotional processing mechanisms. Zhou et al. (2023) developed an EEG-based emotion recognition model, achieving 96.47% accuracy in identifying emotional states, including trust. Joy-type music transformed trust emotions into surprise ones, suggesting that music induces emotional state transitions rather than enhances them. Yüksel et al. (2023) found that cochlear implant recipients reported significantly higher trust levels in response to personally meaningful music than normal hearing controls.

Human-System/Organization Trust (*n* = 2) indicated potential positive patterns through appropriately designed audio characteristics. Schmidmaier et al. (2020) found that ascending melodies were rated more trustworthy than descending melodies, and major mode was more trustworthy than minor mode in technology interactions. Meng et al. (2023) demonstrated that the pleasantness of store music enhanced customer engagement, indirectly increasing customer trust in the store through mediation.

This conceptual mapping reveals that research activity varies substantially across relationship types, with differential methodological approaches and outcome patterns. The distribution of studies across five trust categories indicates broad but sparse coverage of potential music-trust applications, highlighting the need for systematic investigation of context-specific mechanisms, boundary conditions, and methodological standardization before any practical applications can be recommended.

Taken together, these findings indicate that music does not uniformly enhance trust, but rather produces positive, mixed, null, and negative effects depending on contextual, relational, and methodological factors. The presence of null and negative results underscores the importance of identifying boundary conditions under which music may support—or fail to support—trust formation, thereby providing important constraints for the interpretation and future testing of the proposed theoretical framework.

#### 2.2.3 Research gaps and methodological development needs

Landscape mapping reveals critical gaps constraining current understanding and highlights priorities for systematic investigation in this emerging field.

##### 2.2.3.1 Measurement validity concerns

The most significant limitation involves confusion between trust and trustworthiness, with five studies measuring perceived trustworthiness rather than actual trust behavior. This distinction is crucial since perceiving someone as trustworthy does not necessarily translate into trusting behavior involving personal vulnerability (Mayer et al., 1995). Additionally, measurement dissociations emerged across studies employing both self-report and behavioral measures, with musical interventions showing inconsistent effects across different assessment approaches (Evans and Revelle, 2008).

##### 2.2.3.2 Sample and generalizability constraints

Population homogeneity severely limits generalizability, heavily relying on university student samples and predominantly Western populations (Henrich et al., 2010). This is particularly problematic given that musical synchrony may signal conformity in individualist cultures versus harmony in collectivist ones (Markus and Kitayama, 1991). Cultural constraints represent a critical gap, as most studies cannot address how collectivist versus individualist orientations moderate music-trust relationships (Boer and Fischer, 2013).

##### 2.2.3.3 Temporal and ecological validity issues

Most interventions are brief (30–45 min) with immediate post-intervention measurement, lacking long-term follow-up to determine whether music produces lasting trust changes or merely temporary effects (Shadish et al., 2002). Ecological validity concerns arise from artificial laboratory settings that lack contextual factors in real-world trust situations, where decisions have genuine consequences and develop over extended periods (Bronfenbrenner, 1977).

##### 2.2.3.4 Theoretical underdevelopment

Few studies directly tested proposed mechanisms through mediation analysis (Hayes, 2017), leaving unclear whether music influences trust through emotional contagion (Hatfield et al., 1994), synchrony (Hove and Risen, 2009), cognitive load (Sweller, 1988), or other pathways. Most critically, studies have not systematically investigated how intervention characteristics, individual factors, and contextual demands interact to determine the trust-building efficacy of music.

These limitations highlight the preliminary nature of current evidence and underscore the need for more rigorous investigation of underlying mechanisms. While patterns suggest potential influences, definitive causal claims await systematic experimental manipulation of proposed neurobiological pathways. Given the limited direct evidence linking music and trust, the next section adopts a convergence approach by examining shared neurobiological foundations to identify potential pathways through which music might influence trust formation.

## 3 Convergence analysis and theoretical framework development

Given the limited direct evidence linking music and trust, this section presents a hypothesis-generating theoretical framework developed through an exploratory convergence analysis across separate neurobiological literatures. Importantly, this section is distinct from the preceding scoping review and does not synthesize direct empirical evidence on music–trust interactions but instead integrates findings from the broader music and trust literatures to identify potential areas of overlap. We first establish our theoretical foundation using the neuropsychoeconomic T-R-U-S-T framework (Krueger and Meyer-Lindenberg, 2019). We then examine potential convergence patterns across three complementary domains: neurochemical mechanisms, functional neuroimaging, and electrophysiological markers. We conclude by synthesizing these findings into the Preparatory Optimization Hypothesis as a framework for future empirical investigation.

### 3.1 Theoretical foundation and network architecture

We conducted an exploratory synthesis of potential convergence patterns across neurochemical systems, large-scale brain networks, and electrophysiological markers to develop theoretical hypotheses for future research. Our methodology began with established findings from trust research across these three domains, identifying key systems with demonstrated relevance to trust processes. We then systematically examined the much richer music literature, demonstrating superior evidence quality with extensive meta-analytic support. We used these robust meta-analyses and key literature findings to assess domain overlap. This trust-first approach leveraged the superior methodological rigor of music research to examine convergence patterns with established trust mechanisms. This theoretical development is speculative and requires extensive empirical validation, as it draws from separate music and trust research literatures rather than direct music-trust interaction studies. We prioritize meta-analytic evidence where available, supplemented by single studies when higher-level evidence is unavailable, aiming to generate testable hypotheses rather than establish causal mechanisms (Platt, 1964). In this context, convergence is interpreted as the identification of candidate mechanisms through consistent patterns of overlap across domains, rather than as direct evidence of causal or functional equivalence between music and trust processes.

Our analysis employs the neuropsychoeconomic framework (Krueger and Meyer-Lindenberg, 2019), conceptualizing trust through five core components organized as the T-R-U-S-T acronym. This framework captures trust as a fundamental social dilemma: the tension between [T]reachery (risk of betrayal) and [R]eward (anticipated benefits), creating [U]ncertainty resolved through [S]trategy or [T]rustworthiness evaluations. These components engage four domain-general large-scale brain networks—SAN (e.g., insula, anterior cingulate cortex), associated with betrayal aversion; RWN (e.g., striatum, ventral tegmental area), involved in reward anticipation; DMN (e.g., medial prefrontal cortex, temporal parietal junction), involved in social cognition; and CEN (lateral prefrontal cortex, posterior parietal cortex), involved in strategic planning—that process trust decisions through dynamic interactions (Menon, 2011). The four-network architecture provides a theoretical framework for examining potential music-trust convergence because musical experiences and trust formation emerge from distributed neural system interactions rather than single brain region activity (Bullmore and Sporns, 2009). All proposed relationships require systematic empirical validation through direct music-trust interaction studies.

### 3.2 Hormonal and neurotransmitter mechanisms

We begin our convergence analysis at the neurochemical level, examining how molecular mechanisms may provide the biochemical foundation for potential music-trust relationships. The systematic synthesis analysis of neurochemical systems across music and trust domains reveals complex convergence, contestation, and opposition patterns, with music demonstrating consistently stronger evidence quality across multiple systems (see Table 3 for detailed system-by-system analysis).

**Table 3:** Exploratory and hypothesis-generating neurochemical convergence patterns between music and trust processing.

Neurochemical	Pattern	Evidence and pattern	Mechanistic overlap	Domain differences	Key supporting studies
Cortisol (hormone)	Opposite	Music: Repeated findings show cortisol decreases after music interventions, especially in relaxation and post-stress recovery contexts Trust: Acute social-evaluative stress increases cortisol; high cortisol responders show greater trust or betrayal aversion under stress	Cortisol regulates HPA-axis stress reactivity, influencing vigilance, risk appraisal, and affiliative motivation	Music: Parasympathetic recovery and auditory pleasure reduce HPA output Trust: Elevated cortisol recalibrates decision thresholds under uncertainty	Music: de Witte et al. (2022; meta-analysis), Khalfa et al. (2003), Thoma et al., 2013 Trust: von Dawans et al. (2012), Javor et al. (2020)
Oxytocin (hormone)	Convergent, context-sensitive	Music: Increases in peripheral oxytocin occur after relaxing listening and especially during singing (solo or choral), supporting mood elevation and bonding Trust: Meta-analyses of intranasal oxytocin find small increases in in-group trust; effects depend on trait trust levels and social context	Oxytocin dampens amygdala reactivity, enhances social salience, promotes affiliation, and buffers stress	Music: Oxytocin release is most robust with emotionally positive or socially embedded music Trust: Oxytocin promotes trust in low-risk, in-group settings; effects may vanish or reverse under high uncertainty	Music: Nilsson (2009), Grape et al. (2003), Schladt et al. (2017) Trust: Bakermans-Kranenburg and van IJzendoorn (2012; meta-analysis); Kosfeld et al. (2005), Declerck et al. (2020)
Testosterone (hormone)	Opposite/domain-specific	Music: Passive listening yields mixed results, but live performance reliably increases testosterone, with effects modulated by sex Trust: Exogenous testosterone administration reduces trust in economic exchange games	Testosterone responds to social-evaluative threat and enhances vigilance; interacts with cortisol to modulate status and risk-seeking behavior	Music: Performance contexts trigger competitive arousal and testosterone increases; passive listening effects are weak and sex-dependent Trust: Elevated testosterone reduces affiliative trust, especially with unfamiliar partners	Music: Fukui and Yamashita (2003), Turan et al. (2022) Trust: Bos et al. (2010), Boksem et al. (2013)
Dopamine (neurotransmitter)	Convergent	Music: PET studies show dopamine release in caudate (anticipation) and nucleus accumbens (consummation). TMS studies reveal that DLPFC stimulation increases striatal dopamine and musical pleasure Trust: D₂/D₃ antagonist increases belief volatility in trust games; agonist (bromocriptine) increases investment behavior in women.	Dopaminergic mesocorticolimbic circuitry supports reward prediction and learning through precision-tuned prediction errors.	Music: Dopamine encodes hedonic intensity and musical arousal Trust: Dopamine adjusts decision volatility and learning rate during social uncertainty.	Music: Salimpoor et al. (2011), Ferreri et al. (2019) Trust: Mikus et al. (2023), Bellucci et al. (2020)
Serotonin (neurotransmitter)	Convergent	Music: Enhances emotional regulation and mood stability during pleasant listening Trust: Serotonin precursor supplementation increases trust in economic games	Modulates mood, prosociality, and emotional salience; supports moral evaluation and affective response	Music: Effects arise through aesthetic engagement and affective tone Trust: Supports prosocial trust via moral and emotional reasoning.	Music: Evers and Suhr (2000). Trust: Colzato et al. (2013).

DA, dopamine; DLPFC, dorsolateral prefrontal cortex; D₂/D₃, dopamine D2 and D3 receptors; HPA, hypothalamic–pituitary–adrenal axis; OT, Oxytocin; PET, positron emission tomography; TMS, transcranial magnetic stimulation; T, testosterone.

#### 3.2.1 Core convergence findings

Dopamine emerged as the strongest neurochemical bridge, supporting reward learning and motivational salience in both domains. In music, dopamine release during anticipation and consummation of pleasure is robustly shown via PET and TMS studies (Salimpoor et al., 2011; Ferreri et al., 2019). In trust, D₂/D₃ receptor manipulation alters belief updating and investment decisions, demonstrating domain-specific modulation (Mikus et al., 2023; Bellucci et al., 2020). This convergence suggests a shared mechanism for adapting to uncertainty through reward prediction.

Oxytocin shows conditional convergence. Music reliably increases peripheral oxytocin during socially embedded or emotionally positive experiences (Grape et al., 2003; Schladt et al., 2017), while trust effects are context- and trait-dependent—emerging under low risk but vanishing when threat or distrust is high (Kosfeld et al., 2005; Declerck et al., 2020). This asymmetry highlights music’s consistent affiliative potential versus trust’s conditional sensitivity.

Cortisol and testosterone display inverse domain effects. Music lowers cortisol through relaxation and parasympathetic engagement (de Witte et al., 2022; Thoma et al., 2013), while social stress paradigms used in trust-related contexts are associated with elevated cortisol levels, which may influence risk appraisal during social decision-making (von Dawans et al., 2012; Javor et al., 2020). Testosterone rises during musical performance due to evaluative arousal (Fukui and Yamashita, 2003), but suppresses trust in social exchange by enhancing vigilance and dominance motivation (Bos et al., 2010; Boksem et al., 2013).

Serotonin shows modest convergence. It contributes to mood stabilization in music (Evers and Suhr, 2000) and has been linked to prosocial trust via precursor supplementation in economic games (Colzato et al., 2013). Although limited, this evidence supports a shared role in affect regulation and affiliative behavior.

#### 3.2.2 Domain-specific evidence quality distinctions

Music research suggests consistently stronger methodological rigor across neurochemical studies, including multiple meta-analyses and laboratory replications using convergent biomarkers such as cortisol and dopamine (de Witte et al., 2022; Salimpoor et al., 2011). In contrast, trust-related findings remain more heterogeneous and context-sensitive, with some key hormonal effects—particularly those involving oxytocin—failing to replicate under varying conditions of social risk and individual differences (Declerck et al., 2020). These disparities reflect deeper temporal and contextual distinctions: music allows immediate, externally triggered neurochemical modulation, while trust outcomes rely on longer-term social learning and trait-based variability. Notably, effects in the trust domain often depend on dispositional factors or social ambiguity, making them harder to standardize across experimental designs (Kosfeld et al., 2005; Javor et al., 2020).

#### 3.2.3 Integration with Preparatory Optimization Hypothesis

The data support a conditional convergence model in which music is a preparatory enhancer of trust-relevant neurochemistry. Unlike trust, which is shaped by social uncertainty and context-dependent oxytocin effects, music reliably induces cortisol reduction and socially mediated oxytocin increases, especially through emotionally positive or group-based activities (Schladt et al., 2017; Thoma et al., 2013). Given that oxytocin has been linked to trust primarily when social cues are positive and perceived risk is low (Declerck et al., 2020), music’s capacity to establish such conditions may functionally prime affiliative circuits before trust decisions occur.

This temporal complementarity—between music’s rapid, phasic modulation of neurochemical systems and trust’s gradual, integrative development—suggests that music may compensate for the unreliability of direct neurochemical interventions in trust. Dopaminergic and serotonergic convergence further support this preparatory model by enabling mood regulation and prediction-error precision, which influence the willingness to engage in trust behaviors (Colzato et al., 2013; Bellucci et al., 2020). While this analysis focused on neurochemicals with established relevance to trust, future research should explore underexamined candidates such as GABA, acetylcholine, and vasopressin for their potential roles in music-trust integration.

### 3.3 Functional neuroimaging mechanisms

While neurochemical mechanisms provide the molecular foundation for music–trust convergence, functional neuroimaging reveals how these biochemical processes organize into coordinated brain network activity. The exploratory synthesis analysis incorporates convergent neuroimaging findings across music and trust domains, with fMRI studies revealing extensive spatial overlap across four core networks: SAN, DMN, CEN, and RWN. These patterns support shared but functionally distinct neurobiological mechanisms (see Table 4 for detailed network-by-network analysis).

**Table 4:** Exploratory and hypothesis-generating brain network convergence patterns between music and trust processing.

Network	Pattern	Evidence and pattern	Mechanistic overlap	Domain differences	Key supporting studies
Reward network	Convergent	Music: Increased activity in ventral striatum and OFC during musical anticipation and peak pleasure Trust: Increased activity in ventral striatum and vmPFC during partner evaluation and investment decisions	Involves mesocorticolimbic dopamine circuitry (VTA–striatal pathway) and OFC/vmPFC for value computation and reward prediction errors.	Music: Shows temporal dissociation between anticipation (caudate) and consummation (nucleus accumbens), reflecting hedonic variability Trust: Tracks value updating from social priors and moral cues; differentiates conditional and unconditional trust	Music: Koelsch (2020; meta-analysis), Salimpoor et al. (2011) Trust: Bellucci et al. (2017; meta-analysis), Delgado et al. (2005)
Salience Network	Convergent	Music: Increased activity and connectivity in anterior insula and dACC during emotional salience, expectancy violations, and plasticity from long-term training Trust: Increased anterior insula activity when facing betrayal risk or social norm violation.	Anterior insula and dACC detect emotionally salient or threatening stimuli and trigger rapid task-set switching.	Music: Activated by aesthetic violations and predictive surprise; modulated by musical expertise Trust: Activated by social ambiguity, betrayal aversion, and norm violations	Music: Koelsch (2020, meta-analysis), Luo et al. (2014) Trust: Bellucci et al. (2017; meta-analysis), Aimone et al. (2014).
Default/mode network	Convergent	Music: Increased connectivity within precuneus, mPFC, and TPJ during autobiographical or nostalgic music listening. Trust: Increased activity and connectivity in dmPFC, TPJ, and precuneus during mentalizing about a partner’s intentions	Supports self-referential cognition, mental-state attribution, autobiographical memory retrieval, and narrative construction.	Music: Engaged by personal preference and autobiographical recall Trust: Engaged during recursive modeling of others’ beliefs and trait-level trust tendencies	Music: Wilkins et al. (2014), Janata (2009) Trust: Sripada et al. (2009), Lemmers-Jansen et al. (2019)
Central/executive network	Convergent	Music: Increased activity in dorsolateral prefrontal cortex (dlPFC) during syntactic violations and working-memory demands Trust: Increased dlPFC activity during strategic trust decisions; frontoparietal connectivity predicts trait-level trust	dlPFC enables top-down cognitive control, rule-based reasoning, and flexible attention reallocation under uncertainty	Music: dlPFC supports structural parsing and memory integration for complex auditory streams Trust: dlPFC regulates strategy-shifting, inhibition, and valuation during ambiguous social choices	Music: Kunert et al. (2015), Schulze et al. (2011) Trust: Aimone et al. (2014), Bellucci et al. (2019)

dACC, dorsal anterior cingulate cortex; dlPFC, dorsolateral prefrontal cortex; dmPFC, dorsomedial prefrontal cortex; mPFC, medial prefrontal cortex; NAcc, Nucleus accumbens, OFC, orbitofrontal cortex; TPJ, temporoparietal junction; vmPFC, ventromedial prefrontal cortex; VTA, ventral tegmental area.

#### 3.3.1 Core convergence findings

The RWN shows the most robust convergence through shared mesocorticolimbic dopaminergic pathways, with parallel engagement of the ventral striatum and medial prefrontal regions across both domains. Music activates these structures during reward anticipation and peak pleasure (Koelsch, 2020; Salimpoor et al., 2011), while trust engages them during partner evaluation and investment behavior (Bellucci et al., 2017; Delgado et al., 2005). These findings highlight domain-general reward prediction and value computation mechanisms.

SAN convergence centers on the anterior insula’s role in salience detection. In music, anterior insula and dACC are activated by emotional salience and expectancy violations, with effects amplified by musical training (Koelsch, 2020; Luo et al., 2014). In trust, the anterior insula is similarly activated during betrayal risk and norm violation, reflecting its role in monitoring social ambiguity (Bellucci et al., 2017; Aimone et al., 2014). Both domains rely on this network to prioritize meaningful stimuli under uncertainty.

DMN engagement shows consistent overlap but domain-specific emphasis. In music, increased connectivity in the mPFC, TPJ, and precuneus supports autobiographical memory retrieval and personal meaning during emotionally significant listening (Wilkins et al., 2014; Janata, 2009). In trust, these same regions are recruited for mentalizing and modeling a partner’s intentions or reputation (Sripada et al., 2009; Lemmers-Jansen et al., 2019). The shared reliance on self-referential and perspective-taking processes underpins both aesthetic and social cognition.

CEN activation reveals convergent recruitment of the dorsolateral prefrontal cortex (dlPFC), though with different cognitive demands. In music, the dlPFC is activated during syntactic violation detection and musical working memory (Kunert et al., 2015; Schulze et al., 2011). In trust, the same region supports strategic decision-making under uncertainty, with trait-level dlPFC connectivity predicting trust propensity (Aimone et al., 2014; Bellucci et al., 2019). This reflects shared top-down control functions tailored to different task demands.

#### 3.3.2 Domain-specific distinctions

Temporal dynamics reveal systematic functional complementarity. Trust networks demonstrate sustained engagement during social evaluation and belief updating (Bellucci et al., 2017), while music networks operate through rapid, phasic activation during emotionally salient events (Salimpoor et al., 2011). Within the SAN, anterior insula activity in music is often transient and tied to aesthetic violations (Koelsch, 2020), whereas in trust, its activity is sustained during norm monitoring and betrayal anticipation (Aimone et al., 2014).

DMN and CEN also show domain-specific emphasis. Music preferentially engages the DMN during autobiographical recall (Wilkins et al., 2014), while trust recruits the same network for mentalizing and reputational modeling (Lemmers-Jansen et al., 2019). In the CEN, music emphasizes syntactic parsing and auditory working memory (Schulze et al., 2011), whereas trust tasks rely on cognitive control and valuation integration under uncertainty (Bellucci et al., 2019). These patterns reflect how the same neural substrates are flexibly engaged depending on context and cognitive load.

#### 3.3.3 Integration with Preparatory Optimization Hypothesis

The systematic functional complementarity supports the Preparatory Optimization Hypothesis through coordinated network engagement, where music may prepare and optimize neural states for subsequent trust formation rather than directly mimicking trust mechanisms. Convergent activity of mesocorticolimbic reward circuitry is consistent with this model: music engages these pathways through affective anticipation (Salimpoor et al., 2011), while trust engages them for social value computation (Delgado et al., 2005). Similarly, anterior insula activity during aesthetic surprise in music (Koelsch, 2020) parallels its involvement in social risk detection during trust decisions (Aimone et al., 2014). Music-induced activity of memory and executive networks (Wilkins et al., 2014; Kunert et al., 2015) may be associated with neural processes that are also implicated in social-cognitive functions relevant to trust, although such overlap does not establish a causal relationship (Poldrack, 2006).

### 3.4 Electrophysiological mechanisms

Building on neuroimaging evidence of large-scale network coordination, electrophysiological methods such as EEG and ERP provide temporally precise markers of how music and trust processes unfold in real time across the brain. The exploratory synthesis analysis reveals systematic temporal convergences between music and trust across multiple frequency bands and event-related components, with four electrophysiological markers demonstrating convergent neural oscillations and a shared three-stage processing architecture: early detection (0–200 ms), middle evaluation (200–400 ms), and late integration (400 + ms) (see Table 5 for detailed component-by-component analysis).

**Table 5:** Exploratory and hypothesis-generating electrophysiological convergence patterns between music and trust processing.

Component/band	Pattern	Evidence and pattern	Mechanistic overlap	Domain differences	Key supporting Studies
P3/P300	Convergent	Music: Increased P300 amplitude in response to culturally familiar, preferred, or emotionally salient music passages (e.g., familiar instruments or nostalgic melodies) Trust: Increased decision-locked P300 in response to socially salient cues (e.g., voice attractiveness, behavioral consistency, narcissism) that influence trust decisions	Reflects attention allocation, salience detection, and belief updating in response to motivationally relevant stimuli	Music: Driven by memory, structural familiarity, and affective engagement Trust: Driven by salience of social cues influencing decision significance	Music: Arikan et al. (1999), Zhu et al. (2008), de Sá and Pereira (2011) Trust: Yang and Li (2018), Guo et al. (2022), Shang and Liu (2022)
Late positive potential (LPP)	Convergent	Music: Enhanced LPP (400–700 ms) when emotionally congruent music is paired with images, reflecting amplified affective appraisal Trust: Increased LPP (450–650 ms) during decisions to trust high-moral over low-moral faces, highlighting the motivational value of trustworthy cues	Reflects sustained affective evaluation, emotional salience, and higher-order appraisal of auditory or social stimuli	Music: Late positivity enhanced by cross-modal emotional congruence Trust: Modulated by perceived moral traits of the partner at the decision moment	Music: Spreckelmeyer et al. (2006) Trust: Chen et al. (2022)
N2	Convergent	Music: Increased frontal N2 / ERAN; (~160–220 ms) in response to harmonic-syntax violations; effect enhanced by musical expertise Trust: Increased N2 (250–350 ms) during distrust or keep decisions; amplitude tracks social-risk sensitivity and narcissistic traits.	Indicates conflict monitoring and rule-violation detection	Music: Triggered by expectancy violations in tonal grammar Trust: Reflects social conflict when overriding default cooperation or trust norms	Music: Koelsch et al. (2005), Steinbeis et al. (2006) Trust: Wang et al. (2016), Guo et al. (2022)
Beta oscillations (13–30 Hz)	Convergent	Music: Increased beta power (15–25 Hz) during beat anticipation and pitch expectancy violations; strength of modulation depends on predictive cues and musical expertise Trust: Increased decision-locked beta power (14–22 Hz) during trust or distrust decisions; beta magnitude predicts subsequent choice behavior.	Supports sensorimotor prediction, cognitive control, and internal modeling of timing or social rules	Music: Involved in beat perception, rhythm tracking, and motor entrainment Trust: Supports inhibitory control and predictive updating under social uncertainty	Music: Chang et al. (2016), Graber and Fujioka (2020) Trust: Wang et al. (2017), Wang et al. (2021)

LPP, Late Positive Potential; Hz, Hertz; ms, milliseconds; RAN, early right anterior negativity.

#### 3.4.1 Core convergence findings

The strongest convergence appears in attention allocation and context updating mechanisms, with P3/P300 components showing consistent domain overlap. In music, increased P300 amplitude is observed for culturally familiar or emotionally salient passages (Arikan et al., 1999; Zhu et al., 2008), while in trust, P300 has been linked to decision-making influenced by salient social cues such as partner attractiveness or behavioral consistency (Yang and Li, 2018; Shang and Liu, 2022). These findings suggest shared engagement of motivational relevance and belief-updating systems.

The Late Positive Potential (LPP) reflects sustained emotional evaluation. In music, LPP amplitude increases when emotionally congruent musical excerpts accompany images, indicating affective integration (Spreckelmeyer et al., 2006). In trust, LPP is enhanced when participants choose to invest in high-moral partners, marking the motivational salience of trustworthy cues (Chen et al., 2022). This supports convergence in affective appraisal and outcome significance.

N2 components also show cross-domain alignment. In music, frontal N2/ERAN responses are elicited by harmonic-syntax violations, particularly among individuals with musical training (Koelsch et al., 2005; Steinbeis et al., 2006). In trust, N2 increases during distrust or keep decisions, tracking social-risk sensitivity and internal conflict (Wang et al., 2016; Guo et al., 2022). These effects reflect convergent recruitment of conflict-monitoring systems.

Beta oscillations demonstrate domain-specific functionality with convergent structure. In music, increased beta power occurs during beat anticipation and pitch expectancy violations, scaling with musical expertise (Chang et al., 2016; Graber and Fujioka, 2020). In trust, beta activity rises during decisions to maintain or withhold trust, predicting behavioral outcomes under uncertainty (Wang et al., 2017; Wang et al., 2021). This supports complementary roles for sensorimotor prediction and cognitive control in each domain.

#### 3.4.2 Domain-specific distinctions

Temporal profiles reveal key differences. Music shows sustained processing beyond approximately 600 ms following stimulus onset, sup-porting aesthetic memory integration and structural parsing, while neural responses associated with trust-related decision-making are often observed within approximately 500 ms following stimulus presentation, as part of rapid social evaluation processes (Yang and Li, 2018). Salience and conflict markers such as P300 and N2 show stronger modulation by musical training in the music domain (Koelsch et al., 2005), whereas trust responses are more strongly influenced by dispositional variables, in-cluding social risk sensitivity and partner-specific traits (Guo et al., 2022). These domain-specific modulations reflect divergent task demands: aesthetic elaboration versus social judgment under un-certainty.

#### 3.4.3 Integration with Preparatory Optimization Hypothesis

The electrophysiological convergence supports the Preparatory Optimization Hypothesis through sequential modulation of shared attention, evaluation, and control systems. P3/P300 supports salience detection and belief updating, LPP underpins emotional appraisal, N2 supports conflict monitoring, and beta oscillations reflect domain-tailored predictive control. Together, these components form a multi-stage optimization sequence across time. Early detection (0–200 ms) has been linked to rapid stimulus appraisal and expectancy violation sensitivity. Middle evaluation (200–400 ms) recruits context updating and conflict detection systems via P3 and N2 convergence. Late integration (400 + ms) engages sustained affective and cognitive evaluation via LPP and beta activity. This processing architecture suggests that music can pre-tune neural systems critical for trust decisions, while maintaining domain-specific differences in component timing and functional emphasis.

### 3.5 Theoretical synthesis: the Preparatory Optimization Hypothesis as a framework for future research

Having examined potential convergence patterns across neurochemical, neuroimaging, and electrophysiological domains, we now synthesize these findings into a coherent theoretical framework. The convergent evidence across all three levels of analysis supports the development of the Preparatory Optimization Hypothesis as an organizing framework for future empirical investigation.

#### 3.5.1 Convergence patterns across neurobiological domains

Our methodology began with established findings from trust research across neurochemical, neuroimaging, and electrophysiological domains, identifying key systems with demonstrated relevance to trust processes and creating comprehensive supplementary tables documenting all trust studies for each category. We then systematically examined the much richer music literature, demonstrating superior evidence quality with extensive meta-analytic support. We used these robust music findings to assess domain overlap. This trust-first approach leveraged the superior methodological rigor of music research to examine convergence patterns with established trust mechanisms.

The systematic synthesis across neurochemical, neuroimaging, and electrophysiological domains reveals complex patterns of convergence, contestation, and opposition—supporting a preparatory optimization mechanism rather than direct trust induction. Dopamine emerges as the strongest neurochemical bridge, serving reward and learning functions through shared mesocorticolimbic pathways, with music showing robust PET evidence (Salimpoor et al., 2011) and trust demonstrating pharmacological modulation (Bellucci et al., 2020; Mikus et al., 2023). Cortisol and testosterone show systematic opposite effects with overlapping stress regulation functions: music reduces cortisol via relaxation (Thoma et al., 2013), while social stress paradigms used in trust-related contexts are associated with elevated cortisol under social-evaluative stress (von Dawans et al., 2012); testosterone increases during musical performance (Fukui and Yamashita, 2003), but suppresses trust in social exchange (Bos et al., 2010; Boksem et al., 2013).

The four-network architecture (SAN, RWN, DMN, and CEN) suggests extensive spatial convergence with functionally distinct roles. The RWN shows robust overlap through activity of the ventral striatum and prefrontal regions during both hedonic (Salimpoor et al., 2011) and social reward processing (Delgado et al., 2005). SAN convergence centers on anterior insula activity for emotional salience in music (Koelsch, 2020; Luo et al., 2014) and betrayal risk detection in trust (Aimone et al., 2014). DMN specialization contrasts autobiographical memory engagement during music (Wilkins et al., 2014; Janata, 2009) with mentalizing processes during trust (Sripada et al., 2009; Lemmers-Jansen et al., 2019). CEN supports syntactic parsing and musical working memory (Kunert et al., 2015; Schulze et al., 2011) versus strategic reasoning and inhibition in trust (Bellucci et al., 2019; Aimone et al., 2014).

Electrophysiological markers demonstrate convergent neural oscillations and a shared three-stage processing architecture. P3/P300 components show convergence in attentional salience and context updating: in music, via emotional or familiar passages (Zhu et al., 2008); in trust, via socially salient cues (Yang and Li, 2018; Shang and Liu, 2022). LPP indexes sustained affective evaluation in both domains (Spreckelmeyer et al., 2006; Chen et al., 2022). N2 reflects conflict detection during musical expectancy violations (Koelsch et al., 2005) and social risk sensitivity (Guo et al., 2022). Beta oscillations reveal complementary prediction and control mechanisms—musical beat entrainment and expectancy in music (Chang et al., 2016; Gaber, 2020), and trust modulation via decision-locked beta dynamics (Wang et al., 2021).

#### 3.5.2 Preparatory optimization pathways and mechanisms

The Preparatory Optimization Hypothesis proposes coordinated engagement across convergent systems that may work synergistically to create favorable conditions for trust formation through conditional preparation rather than simple convergence. The *stress reduction pathway* operates through cortisol regulation, where music shows consistent therapeutic reduction (Thoma et al., 2013), while trust evaluation elevates cortisol in socially stressful contexts (von Dawans et al., 2012). The *social bonding pathway* is context-dependent. Music reliably increases oxytocin in emotionally positive or group-based settings (Schladt et al., 2017), while trust effects depend on social risk, trait trust, and perceived safety (Kosfeld et al., 2005; Declerck et al., 2020). Music’s capacity to create affiliative, low-risk environments may help compensate for the instability of oxytocin-induced trust in high-threat contexts. The *reward learning pathway* operates through shared dopaminergic mechanisms, with music and trust both activating the ventral striatum and prefrontal reward areas (Salimpoor et al., 2011; Delgado et al., 2005). Finally, the cognitive optimization pathway involves beta oscillation modulation, where sensorimotor beta supports temporal prediction in music (Chang et al., 2016), and decision-locked beta predicts trust behaviors via inhibitory control (Wang et al., 2021).

#### 3.5.3 Predictive extensions of the Preparatory Optimization Hypothesis

The Preparatory Optimization Hypothesis generates five testable predictions that formalize hypothesis-driven extensions of the observed convergence patterns and existing findings. Temporal specificity predicts that musical interventions may influence trust formation via immediate neurochemical modulation that complements longer trust-building processes. Pathway specificity suggests different musical features may differentially engage distinct systems—group singing boosts oxytocin, pleasurable melodies engage shared reward circuits, and rhythm strengthens cognitive control via beta synchronization. Individual difference moderation predicts enhanced optimization effects for individuals with adverse experiences, where music may help compensate for weakened oxytocin-related trust responses. Active engagement specificity emphasizes participatory music over passive listening, reflecting the stronger neurochemical and cognitive effects observed during active engagement (e.g., Schladt et al., 2017; Chang et al., 2016). Network complementarity predicts temporal and functional alignment between music’s phasic network activity and trust’s sustained evaluative processing, supported by shared cascades from salience detection to reward integration and top-down control (Koelsch, 2020; Bellucci et al., 2019), with particular relevance for identifying boundary conditions and context-dependent effects in future empirical studies.

#### 3.5.4 Validation requirements and evidence quality limitations

This theoretical framework remains speculative and requires systematic validation across all levels of analysis. Domain-specific limitations constrain interpretation. Research maturity varies substantially: music neuroscience shows stronger methodological rigor, including meta-analyses and multimodal studies (e.g., Koelsch, 2020; Wilkins et al., 2014), while trust research often lacks replication and demonstrates inconsistent effects (Declerck et al., 2020). Domain-specific differences in neurochemical reliability and trait moderation highlight the need for future work targeting these asymmetries.

The convergence analysis across neurochemical systems, brain networks, and electrophysiological markers supports conditional preparation over direct convergence. Music’s reliable engagement of affective, memory, and control systems may prepare neural environments for affiliative trust interactions. Oxytocin’s sensitivity to social threat in trust, contrasted with music’s stable group-based effects, illustrates the compensatory potential of musical pathways. The Preparatory Optimization Hypothesis should serve as a generative research framework—guiding future empirical studies that integrate domain-specific distinctions, opposite effects, and temporal complementarity identified throughout this analysis.

## 4 Research priorities and conclusions

This section synthesizes our scoping review findings to propose guiding priorities for systematic investigation in this emerging field. Rather than offering an exhaustive roadmap, we highlight representative gaps, methodological considerations, and candidate frameworks that can inform the next generation of research on music–trust relationships.

### 4.1 Research landscape synthesis and critical gaps

Our scoping review reveals an emerging field characterized by sparse and heterogeneous research ac-tivity spanning 15 studies across 35 years. Research distribution across five trust relationship types—Human-Human (*n* = 4), Human-Robot (*n* = 4), Human-Professional (*n* = 3), Human-Self (*n* = 2), and Human-System/Organization (*n* = 2)—indicates broad but limited coverage of potential music–trust domains. The field lacks systematic investigation, standardized methodologies, and theoretical coherence, representing a preliminary research landscape in need of foundational development. Importantly, the relative scarcity of direct music-based studies should not be interpreted as evidence that music lacks trust-related effects, but rather as indicating that direct tests of music as a whole remain less developed than adjacent mechanism-focused literatures, particularly work on synchrony.

Methodological heterogeneity is a defining feature of current research. Studies employ diverse musical interventions, trust measurement approaches, and experimental designs, limiting the comparability of findings and the ability to draw robust conclusions. This diversity reflects the exploratory nature of the field but also highlights the urgent need for theoretical integration and methodological standardization. The exploratory Preparatory Optimization Hypothesis offers one possible organizing framework but remains **a** hypothesis-generating and entirely speculative framework and requires substantial empirical validation before any mechanistic claims can be substantiated.

Several critical research gaps constrain current understanding. How do cultural differences in musical meaning and social trust orientations influence neurobiological responses to musical interventions? The current evidence base, drawn almost exclusively from Western populations, limits understanding of how proposed mechanisms generalize across cultural contexts in which both music and trust may carry fundamentally different meanings. How do clinical populations with altered trust processing respond to musical interventions? Early evidence suggests that individuals with trauma histories, autism spectrum conditions, or social cognition impairments may require tailored approaches. Finally, what are the long-term effects of validated musical trust interventions? Most current findings concern immediate responses, but assessing sustained effects requires longitudinal studies tracking behavioral and neurobiological change over time.

Importantly, the current evidence base is heterogeneous and context-dependent, with studies reporting positive, mixed, null, and negative effects across different domains. These findings suggest that the potential influence of music on trust is not uniform, but rather depends on specific contextual, relational, and methodological factors. Accordingly, the Preparatory Optimization Hypothesis should be interpreted as a conditional and hypothesis-generating framework that requires careful empirical validation, particularly with respect to identifying boundary conditions under which music may support—or fail to support—trust formation.

### 4.2 Essential research priorities and methodology

Multi-measure validation studies represent a top priority. These should combine behavioral trust games with self-report instruments to determine which trust dimensions are influenced by music (Campbell and Fiske, 1959; Berg et al., 1995). Longitudinal effectiveness trials with follow-ups at 1 week, 1 month, and 3 months are necessary to evaluate whether effects are enduring or transient. Cross-cultural studies should examine how individualist versus collectivist orientations moderate music–trust relationships (Henrich et al., 2010), particularly investigating whether musical synchrony signals conformity or harmony depending on cultural context.

Integrated multi-level studies should concurrently assess hormonal (cortisol, oxytocin), neuroimaging (four-network architecture), and electrophysiological (P3, LPP, N2, and beta) responses. Future studies should also directly compare music-based paradigms with non-musical component-based paradigms, especially synchrony-based designs, to clarify which elements of musical engagement are most relevant for trust formation. This design would test whether the Preparatory Optimization Hypothesis has empirical support. Mediation analysis should be employed to determine whether trust effects are driven by stress reduction, social bonding, reward enhancement, or cognitive regulation mechanisms—or whether no such relationships exist (Hayes, 2017). In addition, research should prioritize the mapping of individual difference moderators such as genetic markers, baseline network connectivity, or clinical variables to determine responsiveness and boundary conditions. Current work lacks systematic attention to these critical factors.

Methodological development is also essential. Priorities include: (1) standardized musical stimuli to manipulate specific features (e.g., rhythm, harmony, familiarity); (2) validated and culturally adaptable trust measurement tools; (3) open-access research templates with appropriate control designs; (4) pre-registered power analyses for high-variance constructs; and (5) data-sharing frameworks to enable replication and cumulative knowledge building.

### 4.3 Implementation framework and systematic research programs

We propose a phased implementation framework to guide future research, beginning with laboratory validation and progressing toward ecological application. Phase 1 (Years 1–2) should focus on basic experimental validation under controlled conditions using multiple measurement approaches and diverse participant samples. Phase 2 (Years 2–4) should pursue mechanistic investigations of neurobiological pathways and formal testing of theoretical models. Phase 3 (Years 4–5) should evaluate boundary conditions and ecological validity, exploring real-world contexts only if core effects are empirically demonstrated. Phase 4 (Years 5+) can evaluate translational applications in clinical or organizational contexts, contingent on prior empirical validation. Effective implementation will require interdisciplinary collaboration across neuroscience, psychology, music therapy, and adjacent domains. The field’s progress depends on moving beyond exploratory case studies toward cumulative, multi-level research designs that systematically test theoretical predictions and causal mechanisms. This scoping review provides an initial map of direct music-based research on trust within an emerging domain. By defining key priorities and proposing a framework for systematic investigation, we aim to support a research trajectory that can advance rigorous, evidence-based understanding of how music may influence trust, cooperation, and real-world social connection. Rather than concluding that music lacks trust-related effects, the present review highlights the relative scarcity of direct studies and the need to integrate music-based paradigms with adjacent mechanism-focused work, including research on synchrony. By situating music as a potential neuromodulatory primer for trust formation, this review contributes to music psychology by extending its explanatory scope beyond emotion and prosociality to include foundational social-cognitive processes such as trust.

## Data Availability

The original contributions presented in the study are included in the article/supplementary material, further inquiries can be directed to the corresponding authors.
